# The central role of peripheral nodes in directed network dynamics

**DOI:** 10.1038/s41598-019-49537-8

**Published:** 2019-09-11

**Authors:** Edgar A. P. Wright, Sooyeon Yoon, António L. Ferreira, José F. F. Mendes, Alexander V. Goltsev

**Affiliations:** 1 Departamento de Física & I3N, Universidade de Aveiro, 3810-193 Aveiro, Portugal; 2 A. F. Ioffe Physico-Technical Institute, 194021 St. Petersburg, Russia

**Keywords:** Complex networks, Nonlinear phenomena, Phase transitions and critical phenomena

## Abstract

Many social, technological, and biological systems with asymmetric interactions display a variety of collective phenomena, such as opinion formation and synchronization. This has motivated much research on the dynamical impact of local and mesoscopic structure in directed networks. However, the unique constraints imposed by the global organization of directed networks remain largely undiscussed. Here, we control the global organization of directed Erdős–Rényi networks, and study its impact on the emergence of synchronization and ferromagnetic ordering, using Kuramoto and Ising dynamics. In doing so, we demonstrate that source nodes – peripheral nodes without incoming links – can disrupt or entirely suppress the emergence of collective states in directed networks. This effect is imposed by the bow-tie organization of directed networks, where a large connected core does not uniquely ensure the emergence of collective states, as it does for undirected networks.

## Introduction

**Figure 1:**
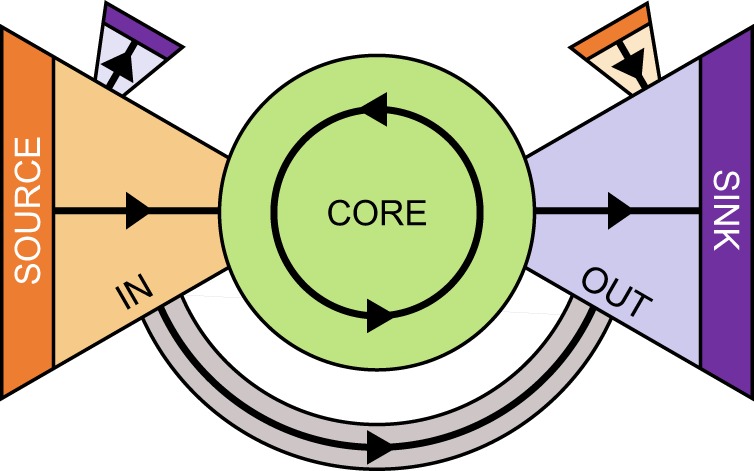
Bow-tie architecture of a directed network, composed of a core and its periphery. The CORE is the largest strongly connected component (largest set of nodes reachable from each other through a sequence of directed links). The periphery comprises the IN and OUT components – the sets of nodes in sequences of directed links leading into and out of the CORE respectively^1–3,43^ – and a hierarchy of tendrils (such as those connecting into and out of the OUT and IN components, respectively) and tubes (directly connecting the IN and OUT components)^36^. As indicated by the arrowheads, the overall connectivity of this architecture is feedforward, from SOURCE nodes (IN nodes without in-links) to SINK nodes (OUT nodes without out-links).

A general strategy for characterizing the global organization of directed networks was first applied to the WWW, as it existed in 1998^1^, revealing the bow-tie architecture schematically depicted in Fig. 1. The core of this architecture is the network’s largest strongly connected component, and all remaining components – the periphery – are hierarchically defined in relation to the core. As a consequence of this organization, the overall connectivity of the periphery is feedforward, directed from nodes without incoming links – sources – to nodes without outgoing links – sinks. Here, we study how these unique structural features of bow-tie architectures constrain collective behavior in the Kuramoto and Ising models on directed ER networks, and discuss the implications of our findings for systems with such features.

Early theoretical work provided tools for determining the structural features of uncorrelated random complex networks with arbitrary degree distributions^2^, and analytical calculations of the relative sizes of bow-tie components were reported shortly thereafter^3^. Since then, applied work has further unravelled the relationship between the bow-tie architecture of directed networks and the functioning of systems abstracted onto them. Financial networks have been employed to study flows of ownership^4^, debt^5^ and risk^6^ between financial actors, revealing their topologically-determined roles. Similarly, the infectious potential of individual nodes in animal trade networks has been determined by their classification within the bow-tie topology of these networks^7,8^. This architecture is also common in biological networks^9^, and has been reported in gene regulatory networks^10^, metabolic networks^11^, and neuronal networks^12,13^.

Motivated by the existing interdisciplinary body of knowledge on bow-tie architectures, the present work seeks to understand the dynamical impact of their unique structural features. Existing investigations of directed networks have largely focused on local and mesoscale structural features such as the fraction of reciprocal links, correlations between in-degree and out-degree, and clustering/community structure^14–16^, among other heterogeneities. While the interplay between local/mesoscale structure and collective phenomena has been demonstrated in technological, biological, and social directed networks^17–19^, the role of the global organization of directed networks in collective phenomena remains largely undiscussed. Here, we consider a simple toy model based on directed Erdős–Rényi (ER) networks, which are both free of potentially confounding structural heterogeneities – since each link is present with the same probability – and their bow-tie structure can be controlled with a single parameter – the mean number of incoming links – as discussed in the following section.

The features captured by our toy model are generally present in real directed networks. Consider the following hand-picked examples: (i) a 2010 snapshot of the Twitter follow network^20^, (ii) the visual motion detection circuit of the common fruit fly^21^, and (iii) the chemical connectome of the *C*. *elegans* roundworm^22^. Our analysis of the bow-tie-architecture of networks (i), (ii), and (iii) reveals that 14%, 32%, and 2% of their nodes are sources, respectively, compared to the 80%, 44%, and 91% of nodes in the corresponding strongly connected cores. Given the feedforward nature of bow-tie architectures (cf. Fig. 1), it follows that sources can in principle influence core dynamics, provided the dynamics of the system are local and pairwise (along links). In fact, if sources make up the entire IN component, they will act as a field on some subset of CORE nodes, with dynamics differing in the choice of dynamical parameters, link properties, and initial conditions. This relatively broad class of dynamics^23^ applies to the *C*. *elegans* connectome, where sources are almost entirely sensory neurons, synaptic strength is weighted, and the entire connectome is optimized to minimize wiring cost^12^. In general, source nodes may also have distinct internal dynamics, potentially capturing specific functional behavior, such as the tweet-sharing activity of notable source nodes in the Twitter follow network, e.g. the Dalai Lama and Eminem (at the time of writing), who have millions more followers than the average user^20^ and may, for example, be more selective about the content they share, or indeed have a different impact depending on the emotional and cognitive content of their tweets.

Among all relevant dynamics, we focus our attention on the Kuramoto model of synchronization. The dynamics of this seemingly simple model have been studied extensively on directed networks^24,25^, under the influence of external fields^26,27^, and subsequently applied to model and study synchronization in real biological networks, including the effects of jet lag on the suprachiamastic nucleus^28^, and the dynamical states of waking- and sleeping-state functional brain networks^29^. Despite its success, the Kuramoto model has yet to be analyzed in the context of bow-tie architectures. In addition, the ferromagnetic Ising model – a stochastic spin model with the same critical exponent as the Kuramoto model – has also been investigated in directed ER networks^30^, including the effects of interfacial noise^31^. Interestingly, the authors of^30^ revealed that the ferromagnetic phase transition occurs above the percolation transition (emergence of the CORE) as a function of increasing mean out-degree (mean number of out-links). By contrast, the results obtained for the Ising model on undirected networks show that the zero-temperature ferromagnetic phase transition and the network’s percolation transition occur at the same critical mean degree^32^. In light of this discrepancy, we also investigate how bow-tie architectures impact ordering in the ferromagnetic Ising model, compared to synchronization in the Kuramoto model. In this way, the present work aims to bridge existing gaps in the literature, and highlight the unique structural constraints imposed on the emergence of collective phenomena in directed complex networks with bow-tie architectures.

## Results

### Structure

While the global organization of directed networks (cf. Fig. 1) is characteristically feedforward (from sources to sinks), the overall connectivity of the core is feedback. On the one hand, CORE nodes can both dynamically adjust to and influence the state of their neighbors (through local pair-wise interactions), but on the other, they are subject to the influence of sources. Here, we study how the number of SOURCE and CORE nodes, and the inter-connectivity of the IN and CORE components affect the balance between the core’s ability to support collective dynamics (through feedback) and the influence of sources. To this end, we consider a simple toy model based on directed Erdős–Rényi (ER) networks, where the structural features of interest are determined by the mean in-degree $\langle {q}_{in}\rangle$^3^ (i.e. the mean number of incoming links), starting with the emergence of the core in the network at $\langle {q}_{in}\rangle =1$ (percolation point).

When determining how sources influence the collective dynamics of the core, we must consider how CORE nodes connect to each other, the number of SOURCE nodes relative to that of CORE nodes, and how the former connect to the latter. Connections from SOURCE to CORE nodes may be direct (through direct links) or indirect (through paths to other IN nodes that are themselves directly connected to CORE nodes). For any directed network where the IN component is entirely composed of SOURCE nodes, the dynamics of the SOURCE nodes are formally equivalent to external fields, which act directly on any number of CORE nodes. In the presence of indirect connections from SOURCE nodes to CORE nodes, the above-mentioned equivalence may no longer apply, depending both on the intra-connectivity of the IN component and the dynamical rules themselves. Here, we simply focus on the fraction of SOURCE nodes – *N*_SOURCE_/*N* – relative to the fraction of CORE nodes – *N*_CORE_/*N* – and on the number of links between the IN and CORE components – *L*_IN–CORE_/*L* – in ER networks with *N* nodes and $L=N\langle {q}_{in}\rangle$ links.

**Figure 2:**
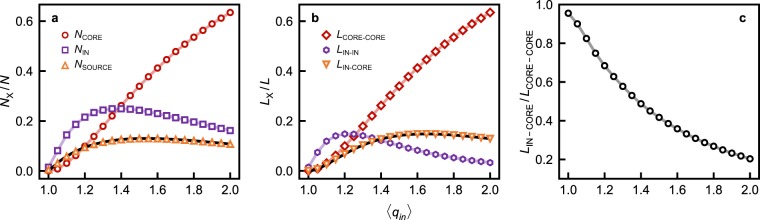
Structural features of directed ER networks as a function of the mean in-degree $\langle {q}_{in}\rangle$: (**a**) The number of nodes *N*_X_ in set X, one of SOURCE (up triangles), IN (squares), and CORE (circles), normalized to the total number of nodes *N* in the network. (**b**) The total number of links between IN nodes *L*_IN–IN_ (hexagons), from IN to CORE nodes *L*_IN–CORE_ (down triangles), and between CORE nodes *L*_CORE–CORE_ (diamonds), normalized to the total number of links *L* in the network. (**c**) The ratio *L*_IN–CORE_/*L*_CORE–CORE_ (see equation (1) and the accompanying text for further detail). The solid lines in (**a**) and (**b**) represent theoretically calculated quantities. See the Methods section for additional information.

Above the percolation point – $\langle {q}_{in}\rangle =1$ – the fraction of CORE nodes – *N*_CORE_/*N* – increases monotonically with $\langle {q}_{in}\rangle$, as shown in Fig. 2(a), and eventually comprises the majority of the network’s nodes. However, the fraction of SOURCE nodes – *N*_SOURCE_/*N* – remains of the same order of magnitude as the fraction of CORE nodes – *N*_CORE_/*N* – for $1\le \langle {q}_{in}\rangle \le 2$, as confirmed both through simulations and analytic calculations, and shown in Fig. 2(a). The influence of this finite fraction of SOURCE nodes is ultimately exerted through a fraction of IN-CORE links – *L*_IN–CORE_/*L* – which is also of the same order of magnitude as the fraction of CORE-CORE links – *L*_CORE–CORE_/*L* – i.e. non-vanishingly small, as shown in Fig. 2(b). Individual CORE nodes adjust their state both through links from other CORE nodes, and from IN nodes, so that their dynamics are partly determined by the balance between their external in-degree ${q}_{in}^{{\rm{ext}}}$ (the number of in-links from IN nodes) and their internal in-degree ${q}_{in}^{{\rm{int}}}$ (the number of in-links from other CORE nodes), where ${q}_{in}={q}_{in}^{{\rm{ext}}}+{q}_{in}^{{\rm{int}}}$. For a typical (average) CORE node, we may consider the ratio between the average external and internal in-degree as a measure of the node’s susceptibility to the influence of the IN component. This is equivalent to analyzing the density of the inter-connectivity between IN and CORE components, relative to the density of the CORE’s own connectivity. Symbolically,

1 $$\frac{\langle {q}_{in}^{{\rm{ext}}}\rangle }{\langle {q}_{in}^{{\rm{int}}}\rangle }=\frac{{L}_{{\rm{IN}}\mbox{--}{\rm{CORE}}}}{{L}_{{\rm{CORE}}\mbox{--}{\rm{CORE}}}},$$

where *L*_IN–CORE_ is the total number of IN-CORE links (external links), and *L*_CORE–CORE_ the total number of CORE-CORE links (internal links).

Looking at the data in Fig. 2(b), we see that although *L*_IN–CORE_ varies non-monotonically with $\langle {q}_{in}\rangle$, *L*_CORE–CORE_ increases monotonically, causing the ratio in equation (1) to decrease monotonically, and suggesting that the average CORE node becomes less susceptible to the influence of the IN component with $\langle {q}_{in}\rangle  > 1$. The CORE becomes larger as it becomes more densely connected, while the IN component shrinks and becomes less densely connected to the CORE, decreasing the susceptibility of CORE nodes to the influence of SOURCE nodes (direct or indirect). However, while the susceptibility of a typical CORE node to the influence of SOURCE node dynamics decreases with $\langle {q}_{in}\rangle$, both the number of SOURCE nodes and the number of IN-CORE links are non-negligible for $1\le \langle {q}_{in}\rangle \le 2$, and may therefore significantly impact the collective dynamics of the CORE given specific dynamical rules and parameters. In addition, the brief survey of directed networks presented in Table 1 below suggests that these structural features are equally significant (non-vanishingly small) in a broad range of directed networks.

**Table 1:** Structural features of real and synthetic directed networks with *N* nodes, mean in-degree $\langle {q}_{in}\rangle$, and $L=N\langle {q}_{in}\rangle$ links: *N*_SOURCE_ is the number of SOURCE nodes, *N*_CORE_ the number of CORE nodes, *L*_IN–CORE_ the number of IN-CORE links, and *L*_CORE–CORE_ the number of intra-CORE links.

Network	*N*	〈*q*_*in*_〉	*N*_SOURCE_/*N*	*N*_CORE_/*N*	*L*_CORE–CORE_/*L*	*L*_IN–CORE_/*L*_CORE–CORE_
Gene Regulatory Network^44^	2.9 × 10^3^	2.94	0.06	0.10	0.23	0.13
Twitter Follow Network^20^	41.7 × 10^6^	17.6	0.14	0.80	0.95	0.04
Optic Medulla Circuit^21^	1.8 × 10^3^	9.44	0.32	0.44	0.12	0.17
Erdős–Rényi Network	1 × 10^5^	1.50	0.13	0.34	0.34	0.42

### Dynamics

Next, we investigated how the above-discussed structural features impact the emergence of synchronization and magnetization in the Kuramoto and Ising models, by controlling the corresponding dynamical parameters – the coupling strength *K* and the temperature *T* – along with the structural control parameter – the mean in-degree $\langle {q}_{in}\rangle$.

#### Kuramoto Model

The Kuramoto model is a phenomenological model of synchronization between coupled phase oscillators^25,33–35^. On a directed network with *N* nodes, the instantaneous change in the phase *θ*_*n*_ of oscillator *n* is governed by:

2 $${\dot{\theta }}_{n}={\omega }_{n}+K\,\mathop{\sum }\limits_{m=1}^{N}\,{A}_{mn}\,\sin ({\theta }_{m}-{\theta }_{n}),$$

where ${\omega }_{n}$ is the oscillator’s natural frequency, *K* is the coupling strength, and *A*_*mn*_ is the network’s adjacency matrix element, which is 1 if node *m* has an out-link to node *n*, and 0 otherwise. The state of the system is described by the complex order parameter

3 $$z(t)=r(t){e}^{i\psi (t)}=\frac{1}{N}\,\mathop{\sum }\limits_{n=1}^{N}\,{e}^{i{\theta }_{n}},$$

where $\psi (t)$ is the average phase. For sufficiently large *K*, the system becomes partially synchronized: a small finite  fraction of oscillators lock at frequency $\Omega$, such that their individual phases ${\theta }_{n}=\Omega t+{{\rm{c}}}_{n}$ for any phase shift $\psi -\pi /2 < {{\rm{c}}}_{n} < \psi +\pi /2$. The phases of unlocked oscillators simply drift, i.e. they are time-wise uncorrelated with the average phase. The extent of synchronization (ordering) in the system is conventionally described by the amplitude of the complex order parameter, the order parameter

4 $$r=\frac{1}{N}\,\mathop{\sum }\limits_{n=1}^{N}\,\cos \,({\theta }_{n}-\psi )=\sqrt{{[{\rm{Re}}(z)]}^{2}+{[{\rm{Im}}(z)]}^{2}}.$$

By definition, the order parameter in equation (4) is an average over all *N* nodes in the network, so it is easily redefined over a subset X with *N*_X_ nodes (e.g. the CORE), and interpreted as the extent of synchronization over the subset, denoted *r*_X_. In the interest of consistency, this notation will be used throughout the present work for all quantities of interest.

**Figure 3:**
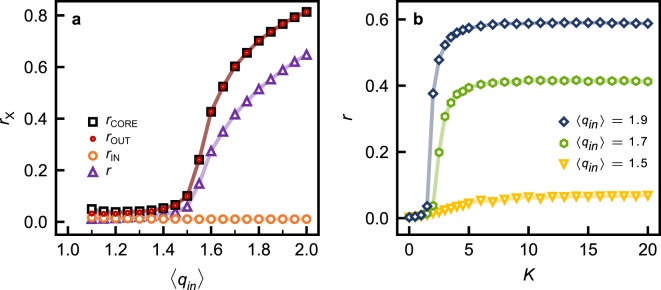
(**a**) Order parameter *r*_X_ as a function of the mean in-degree $\langle {q}_{in}\rangle$, where X is the set of nodes corresponding to the entire network (up triangles) or its IN (circles), CORE (squares) and OUT (dots) components, for *K* = 10 (note the extensive overlap between *r*_CORE_ and *r*_OUT_). (**b**) Order parameter *r* for ER networks with mean in-degree $\langle {q}_{in}\rangle$ equal to 1.5 (down triangles), 1.7 (hexagons) and 1.9 (diamonds) as a function of the coupling strength *K*. For details on the calculation methods see the Methods section.

The large-time average of equation (4) is presented in Fig. 3(a) for different ER networks (as a function of increasing $\langle {q}_{in}\rangle$) and their IN, OUT, and CORE components. Networks with $\langle {q}_{in}\rangle \lesssim 1.5$ are unsynchronized, despite the emergence of the CORE at $\langle {q}_{in}\rangle =1$, whereas networks with $\langle {q}_{in}\rangle \gtrsim 1.5$ are partially synchronized, and synchronization increases with $\langle {q}_{in}\rangle$. The effect of coupling strength *K* on synchronization in different ER networks is presented in Fig. 3(b), where it is shown that the extent of synchronization is limited by the topology of the network when $K\gg 1$.

The data in Fig. 3(a) show that synchronization emerges simultaneously in the network, the CORE, and the OUT component at $\langle {q}_{in}\rangle \simeq 1.5$. This suggests that synchronization is supported by the CORE, as expected from the latter’s feedback connectivity, and is further substantiated by the increase in synchronization with $\langle {q}_{in}\rangle  > 1.5$, as the CORE spans an increasingly larger fraction of the network, and becomes more densely connected. In addition, Fig. 3(a) shows that the IN component is unsynchronized at all values of $\langle {q}_{in}\rangle$, in the limit of large coupling ($K\gg 1$). In this limit, the extent of synchronization in the network is determined by $\langle {q}_{in}\rangle$, as shown in Fig. 3(b). Thus, the overall feedforward connectivity of the network, the absence of synchronization in the IN component, and the increase in synchronization among CORE nodes with $\langle {q}_{in}\rangle  > 1.5$ – as the IN component becomes less densely connected to the CORE – suggest that the IN component may disrupt synchronization in the CORE, and by extension, completely suppress synchronization in the CORE for $1\le \langle {q}_{in}\rangle  < 1.5$. Below $\langle {q}_{in}\rangle =1$, the network does not have a CORE, and synchronization between a finite fraction of the network’s nodes is therefore impossible.

#### Ising Model

The Ising model describes ferromagnetism through the interaction of spins *s*_*n*_ and *s*_*m*_ at sites *n* and *m*. Here, we analyze the time evolution of the dynamics of a spin system in contact with a heat bath at temperature *T*^30^. Each spin *s*_*n*_ experiences the effect of its neighboring spins as a local field

5 $${h}_{n}=\mathop{\sum }\limits_{m=1}^{N}\,{A}_{mn}{s}_{m},$$

and is in a spin up state (${s}_{n}=1$) with probability

6 $${p}_{n}=\frac{1}{1+\exp (\,-\,2{h}_{n}/T)},$$

or in a spin down state (${s}_{n}=-\,1$) with probability $1-{p}_{n}$. The extent of magnetization (ordering) in the system is given by the magnetization per spin site

7 $$m=\frac{1}{N}\,\mathop{\sum }\limits_{n=1}^{N}\,{s}_{n}.$$

**Figure 4:**
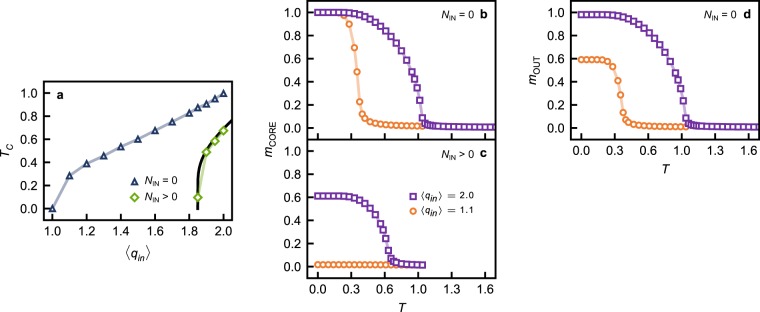
(**a**) Critical temperature *T*_*C*_ in the CORE of directed ER networks with different mean in-degrees $\langle {q}_{in}\rangle$, with (diamonds) and without (triangles) their corresponding IN components. The solid black line was calculated using the mean-field equations reported in^30^. For networks with $\langle {q}_{in}\rangle =1.1$ (circles) and $\langle {q}_{in}\rangle =2.0$ (squares), with – (**c**) – and without – (**b**,**d**) – the corresponding IN components: (**b**,**c**) the magnetization in the CORE (*m*_CORE_), and (**d**) the magnetization in the OUT component (*m*_OUT_) as a function of the temperature *T*. For details on the calculation methods please refer to the Methods section.

Similarly to what is reported here for the Kuramoto model, the authors of^30^ reported a delayed onset of ordering in the zero-temperature ferromagnetic Ising model at $\langle {q}_{in}\rangle \simeq 1.9$. Given the role of the CORE in supporting ordering, and of the IN component in disrupting it (as suggested by the data in Fig. 3), we investigated how magnetization is affected by temperature in directed ER networks, with and without their corresponding IN components. In the absence of the IN component, the CORE of any network with $\langle {q}_{in}\rangle  > 1$ is magnetized at any temperature $T < {T}_{C}$, as shown in Fig. 4(a). In fact, *T*_*C*_ increases monotonically with the CORE’s link density, and is therefore characteristic of the internal connectivity of the CORE. The more densely connected the CORE, the larger the range of temperatures for which it can support magnetization, which is maximal in the limit when $T\to 0$, independently of the CORE’s connectivity, as shown in Fig. 4(b). In the presence of the IN component, the *T*_*C*_ of the CORE is significantly different, and magnetization is disrupted/suppressed, as shown in Fig. 4(a,c), respectively. Although spins in the IN component are at the same temperature as the CORE, they can reduce the range of temperatures at which the CORE supports magnetization, or indeed suppress magnetization altogether, even in the limit where $T\to 0$.

The feedforward organization of the IN component, and the latter’s impact on ordering among CORE nodes with Kuramoto and Ising dynamics hint at the role of SOURCE nodes in limiting the emergence of ordering in the CORE, as does the data in Fig. 4(b,d). Given the overall feedforward connectivity of the bow-tie architecture, we could expect the OUT component to be fully ordered in the absence of the IN component, driven by core dynamics. However, a comparison of Fig. 4(b,d) shows this is not the case. At $\langle {q}_{in}\rangle =1.1$, magnetization is significantly lower in the OUT component than in the CORE. The size of the CORE decreases approaching the network’s percolation point ($\langle {q}_{in}\rangle =1$), while the number of tendrils increases^36^, including those that connect SOURCE nodes to OUT nodes (cf. Fig. 1), potentially explaining the above-mentioned discrepancy.

#### Pair-correlation functions: measuring the dynamical influence of SOURCE nodes

Whether direct or indirect, the influence of SOURCE nodes is experienced by individual CORE nodes as an external field through links from IN nodes. Any strategy to control both of these structural features is heavily dependent on the intra-connectivity of the IN component. For example, removing a SOURCE node may simply create one or more in its place, and removing any number of IN-CORE links does not control for the number of existing SOURCE nodes. Here, we indirectly control these features by simply removing fractions of IN nodes (*f*_IN_) uniformly at random, and calculate the CORE’s response to these structural changes. In the Kuramoto model, this response can be calculated in the rotating frame (at group velocity Ω), using the pair-correlation function

8 $$\begin{array}{rcl}C & = & \frac{1}{N}\,\mathop{\sum }\limits_{i,j=1}^{N}\,[{\langle \cos ({\theta }_{i})\cos ({\theta }_{j})\rangle }_{t}-{\langle \cos ({\theta }_{i})\rangle }_{t}{\langle \cos ({\theta }_{j})\rangle }_{t}]\\  & = & N\,[{\langle {r}^{2}\rangle }_{t}-{\langle r\rangle }_{t}^{2}],\end{array}$$

introduced in^37^, where ${\langle Q(t)\rangle }_{t}=\frac{1}{\tau }\,{\int }_{0}^{\tau }\,dt\,Q(t)$ for some observation time $\tau \gg 1$, for any time-dependent observable *Q*(*t*). In the Ising model, the pair-correlation function is similarly defined

9 $$\begin{array}{rcl}\chi  & = & \frac{1}{N}\,\mathop{\sum }\limits_{i,j=1}^{N}\,[{\langle {s}_{i}{s}_{j}\rangle }_{t}-{\langle {s}_{i}\rangle }_{t}{\langle {s}_{j}\rangle }_{t}]\\  & = & N\,[{\langle {m}^{2}\rangle }_{t}-{\langle m\rangle }_{t}^{2}].\end{array}$$

**Figure 5:**
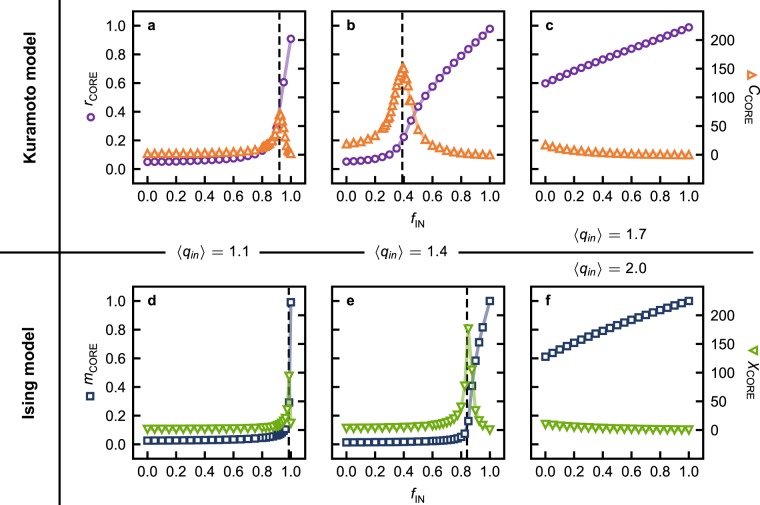
Synchronization *r*_CORE_ (circles) and magnetization *m*_CORE_ (squares) in the Kuramoto – (**a**–**c**) – and Ising – (**d**–**f**) – models, with the corresponding pair correlation functions *C*_CORE_ (up triangles) and *χ*_CORE_ (down triangles), in the CORE of directed Erdős–Rényi networks with mean in-degree $\langle {q}_{in}\rangle$, as a function of the fraction of randomly removed IN nodes *f*_IN_. The values of $\langle {q}_{in}\rangle$ are 1.1 in (**a** and **d**), 1.4 in (**b** and **e**), 1.7 in (**c**), and 2.0 in (**f**). Dashed vertical lines indicate peaks in *C*_CORE_ or *χ*_CORE_. For details on the calculation methods see the Methods section.

The dependence of the synchronization order parameter *r*_CORE_ and *C*_CORE_ on *f*_IN_ is presented in Fig. 5(a,b) for unsynchronized networks, and in Fig. 5(c) for a partially synchronized network, i.e. for networks with $\langle {q}_{in}\rangle  < 1.5$ and $\langle {q}_{in}\rangle  > 1.5$, respectively. Likewise, *m*_CORE_ is also calculated as function of *f*_IN_, and *χ*_CORE_ is used as a measure of the CORE’s response to the influence of the remaining IN component, as shown in Fig. 5(d,e) for unmagnetized networks, and Fig. 5(f) for a partially magnetized network.

In networks with $\langle {q}_{in}\rangle  < 1.5$, synchronization appears only after the removal of a critical fraction ${f}_{{\rm{IN}}}^{C}$ of randomly selected IN nodes, as shown in Fig. 5(a,b). Once the CORE is partially synchronized, the removal of further IN nodes enhances synchronization, similarly to the removal of any fraction of IN nodes from networks with $\langle {q}_{in}\rangle  > 1.5$, where synchronization is linearly enhanced by the removal of randomly selected IN nodes, as shown in Fig. 5(c). The absence of any peaks in *C*_CORE_ for partially synchronized networks and its decaying behavior with *f*_IN_ further suggest that the observed enhancement in synchronization results from more CORE nodes being drawn into the existing synchronized group. In unsynchronized networks, the emergence of synchronization at ${f}_{{\rm{IN}}}^{C}$ is accompanied by a clear peak in *C*_CORE_, signaling the emergence of long-range order that is characteristic of a second order phase transition, in this case from an unsynchronized phase to a synchronized phase, and confirming that synchronization is supported by the CORE and disrupted by the IN component. For networks where $\langle {q}_{in}\rangle  < 1.9$, the removal of a critical fraction of random IN nodes ${f}_{{\rm{IN}}}^{C}$ causes a magnetized phase to emerge in the CORE, as shown in Fig. 5(d,e) by the corresponding peaks in *χ*_CORE_. As further IN nodes are removed, magnetization in the CORE is enhanced, becoming maximal upon the complete removal of the IN component. Similarly, magnetization in the CORE of networks with $\langle {q}_{in}\rangle  > 1.9$ is maximized by removing the entire IN component, as shown in Fig. 5(f), and enhanced by the removal of any fraction of IN nodes. These findings are in agreement with those for synchronization, confirming that the IN component can suppress the emergence of ordered states in the CORE of directed networks, for phenomena as distinct as magnetization and synchronization.

## Discussion

The results presented in the preceding sections show that the global organization of directed networks constrains the emergence of collective phenomena. Specifically, it was shown that the emergence of ordered states in the Kuramoto and Ising models is supported by the CORE and disrupted by the IN component. Here, we discuss how the uncorrelated dynamics of SOURCE nodes are responsible for this effect. By definition, SOURCE nodes do not have in-links, and are therefore unaffected by feedback (cf. Fig. 1), so that SOURCE nodes with randomly distributed initial states remain uncorrelated for all time, acting as a source of noise or fluctuations.

The exact output of the IN component is determined not only by the dynamics of SOURCE nodes, but also by its internal connectivity, which is beyond the scope of this discussion. Nonetheless, it is clear that the number of direct links from SOURCE nodes to the CORE increases with $\langle {q}_{in}\rangle$ (cf. Fig. 2). Additionally, it has been recently shown that, in directed uncorrelated random complex networks, the number of nodes *s* in the finite in-component of any node scales as ${s}^{-3/2}{e}^{-s/{s}^{\ast }}$, where s* is a characteristic parameter that depends on $\langle {q}_{in}\rangle$^38^. This means that only a small number of IN nodes can be reached by traveling backwards from any other IN node, and at least one of these must be a SOURCE node. Above the network’s percolation threshold, the number of SOURCE nodes is always of the same order as the total number of IN nodes. Given the effective absence of reciprocal links and structural correlations that characterize directed ER networks, the above information suggests that paths from individual SOURCE nodes to the CORE are largely disjoint, even when the links to the CORE are not direct.

**Figure 6:**
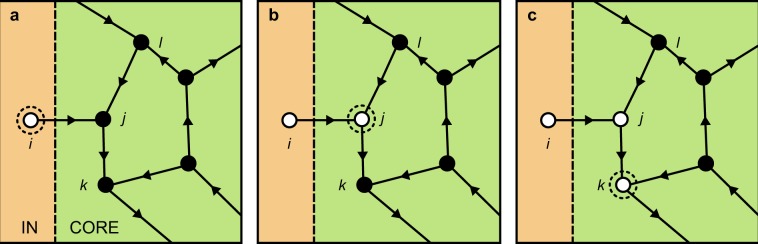
Schematic depiction of how the spin state of an IN node can frustrate the dynamics of a CORE node, at the boundary between IN and CORE components (dashed line). White (hollow) nodes represent spin down states and black (full) nodes represent spin up states. In box (**a**), SOURCE node *i* is linked to a CORE node *j* with a single in-link from another CORE node. As its neighbors *i* and *l* are in opposite spin states, node *j* experiences a local field ${h}_{j}={s}_{i}+{s}_{l}=0$, and is now equally likely to be in a spin up or down state, cf. equation (6). Box (**b**) represents the latter outcome, and box (**c**) a further step with a similar outcome, where node *j* frustrates another CORE node *k*.

Let us now consider the specific case of the Ising model. According to equation (5), each SOURCE node *i* experiences a null local field ${h}_{i}=0$ as a consequence of not having in-links. From equation (6), it then follows that SOURCE nodes are independently and identically found in spin up states (${s}_{i}=1$) and spin down states (${s}_{i}=-\,1$) with probability 1/2, acting as a source of fluctuations that will affect all downstream nodes, at any temperature *T*. In Fig. 5, the CORE is shown to fully magnetize in the absence of the IN component, when $T\to 0$, so that all CORE nodes are in the same spin state. Upon restoring the IN component, and with it ${q}_{in}^{ext}$ links to a given CORE node *j*, the latter will experience fluctuations in its local field *h*_*j*_. If the number of links from CORE nodes ${q}_{in}^{{int}}\le {q}_{in}^{ext}$, the fluctuations will inevitably lead to a configuration of spins where ${h}_{j}=0$ or ${h}_{j} < 0$. Both outcomes will frustrate the dynamics of CORE node *j*, which will change its spin state. While the exact probability of such an event is outside the scope of this discussion, in Fig. 6, we consider a simplified picture, where a single SOURCE node *i* is directly linked to a CORE node *j*, and ${q}_{in}^{int}={q}_{in}^{ext}=1$, a frequent configuration in directed ER networks with $1\le \langle {q}_{in}\rangle \le 2$.

In the above simplified picture, the dynamics of node *j* are frustrated with probability 1/2, as depicted in Fig. 6(a,b). When node *j* is frustrated, it may then frustrate another CORE node *k* in a similar manner, as depicted in Fig. 6(c). Clearly, the fraction of CORE nodes which can be frustrated in this manner is dictated by the internal connectivity of the CORE, the number of links from IN nodes to individual CORE nodes, and the number of SOURCE nodes themselves. Given the similar dependence of magnetization on the above-mentioned structural features identified in the preceding section, frustration presents itself as the putative mechanism behind this dependence, driven by the intrinsic dynamics of SOURCE nodes. When a critical fraction of CORE nodes becomes frustrated, long-range order is broken, suppressing magnetization altogether.

In the Kuramoto model, SOURCE nodes with randomly-distributed natural frequencies act as a source of incoherence: each SOURCE node *i* rotates steadily at its own natural frequency ${\omega }_{i}$, as a consequence of not having in-links, with a phase shift determined by its initial phase (cf. equation (2)). If the coupling strength *K* is sufficiently larger than the width of the frequency distribution $g(\omega )$, any downstream node *j* with a single in-link from node *i* will be driven at frequency ${\omega }_{i}$. A simple stability analysis of equation (2) also shows that node *j* is driven at frequency ${\omega }_{i}$ if the number of in-neighbors rotating at ${\omega }_{i}$ exceeds the magnitude of the remaining in-neighbor’s combined angular velocity. On the one hand, this is demonstrative of the difficulty in predicting the exact output of the IN component to the CORE, other than when the IN component is composed of linear disjoint chains or trees with distinct SOURCE nodes at their root. On the other hand, it reveals that a CORE node *j* can be prevented from joining a group of locked CORE nodes simply by receiving an appropriate number of links from IN nodes. The data presented in Fig. 5 show that the CORE fully synchronizes in the absence of the IN component, and by averaging equation (2) over the CORE it follows that its nodes are rotating at the mean natural frequency $\langle \omega \rangle$. So, upon restoring the IN component, a single SOURCE node *i* rotating at a frequency other than $\langle \omega \rangle$ can prevent a CORE node *j* from joining the synchronized group. Moreover, as ${\omega }_{i}$ is narrowly distributed about $\langle \omega \rangle$, the desynchronizing influence directly experienced by different CORE nodes will also vary, which may compound the desynchronizing effect.

**Figure 7:**
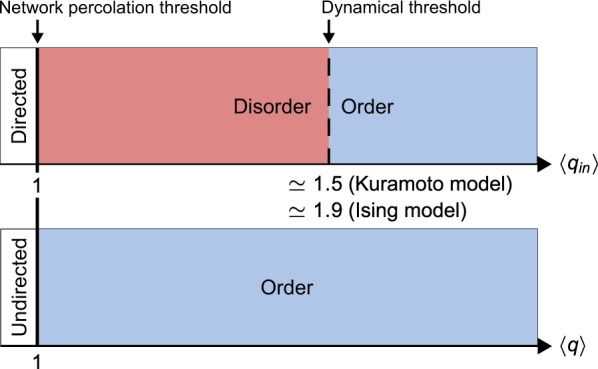
Comparison of the topological restrictions on the emergence of ordered states in the Ising (IM) and Kuramoto (KM) models, in undirected and directed Erdős–Rényi networks, as parameterized by their mean degree $\langle q\rangle$ and mean in-degree $\langle {q}_{in}\rangle$, respectively. The solid vertical line indicates the network’s percolation threshold, and the dashed vertical line the delayed onset of magnetization (IM) and synchronization (KM) relative to the percolation threshold of directed ER networks.

Regardless of the exact output of the IN component, it is clear that SOURCE nodes can directly or indirectly impact the collective dynamics of the CORE. SOURCE nodes are a characteristic feature of the global organization of directed networks, and in this work it was shown that, at least for directed ER networks, when the dynamics of SOURCE nodes are uncorrelated, magnetization and synchronization can be suppressed. This represents an additional constraint when compared to undirected networks, where these ordered states emerge at the network’s percolation threshold, as summarized in Fig. 7.

Given the evident importance of SOURCE nodes in determining the state of the CORE, these findings reinforce their importance for the controllability and resilience of systems abstracted onto directed networks. For example, a synchronized brain network may be desynchronized through the random destruction of synapses, e.g. in Alzheimer’s disease, as the random destruction of links alters the global organization of the network^38^. Similarly, a targeted attack on low in-degree centrality nodes may allow an attacker to exert considerable influence on a network, by controlling the dynamics of newly-created SOURCE nodes. In general, local pairwise dynamics on bow-tie architectures, which may include intrinsic (node-specific), external (field), and stochastic contributions^23^, are mapped to CORE dynamics under the direct or indirect action of the effective field created by SOURCE nodes.

Despite the broad class of dynamics that are susceptible to the influence of SOURCE nodes, the body of work demonstrating bow-tie organization in real systems, and the applications of dynamical models under the action of external fields, any generalizations regarding the dynamics of real systems must be made with care. Firstly, the existence of heterogeneities in the CORE of real networks, such as hubs (scale-free networks) and reciprocal connections, may modify the nature of critical transitions and the values of the critical parameters themselves^32^. However, for particular phase transitions, e.g. percolation, one can also note that structural features such as degree-degree correlations and clustering can change the critical point and critical exponents, but not the nature of the transition^39,40^. Secondly, there are dynamics, e.g. social dynamics, which are more aptly described by agent-based models^19,41,42^. Thirdly, there are further relevant theoretical aspects to consider, such as boundary conditions, the nature of the interactions themselves, and the existence of external driving fields, which are common in real-world systems. In some sense, sources and sinks are a boundary in bow-tie architectures, and the boundary conditions are therefore automatically defined by the dynamics of sources and sinks (regardless of whether these are internally or externally driven). Changing these boundary conditions may produce significantly different collective behavior. For example, if all SOURCE oscillators are assigned the same natural frequency, they may drive synchronization in the CORE at the same frequency, or even cause the emergence of other macroscopic dynamics, analogously to what happens in the Kuramoto model driven by an external field^26,27^. Likewise, different types of interaction can produce different behavior on the same network structure e.g. the antiferromagnetic Ising model displays spin-glass like behavior caused by long loops (cycles)^32^.

In conclusion, we considered a toy model based on directed Erdős–Rényi networks, where the global organization of the network is determined by the mean in-degree $\langle {q}_{in}\rangle$. Considering both Ising and Kuramoto dynamics, we found that the global organization of directed networks constrains the emergence of magnetized and synchronized phases. Unlike in undirected Erdős–Rényi networks, where these collective phenomena emerge at the network’s percolation point, in directed Erdős–Rényi networks, SOURCE nodes with uncorrelated dynamics act to disrupt the collective dynamics of the CORE, delaying the onset of ordered states above the network’s percolation point, where $\langle {q}_{in}\rangle =1$. Magnetization was confirmed to emerge for $\langle {q}_{in}\rangle \gtrsim 1.9$, and synchronization found to emerge for $\langle {q}_{in}\rangle \gtrsim 1.5$. The clear impact of SOURCE nodes, a topological feature only found in directed networks, highlights the need to consider the global organization of directed networks when considering their dynamics, robustness, and controllability. Based on the discussion in the preceding paragraph, we believe that source nodes will produce similar effects in strongly heterogeneous, degree-degree correlated, and clustered networks with a bow-tie architecture, but further investigations are required.

## Methods

The ER toy-model is based on ensembles of 50 directed ER networks with mean in-degree $\langle {q}_{in}\rangle$. Each network is generated from its undirected counterpart by first assigning a direction to all links ($i\to j$, where $i < j$) and then reversing it with probability 1/2. The undirected ER networks were generated by creating links between all possible labeled pairs of $N={10}^{5}$ nodes with probability $2\langle {q}_{in}\rangle /(N-1)$. Note that in this kind of ER network, two nodes are connected by a single directed link, and reciprocal links are absent by construction. Directed ER networks can also be built by forming any possible directed link with probability ~1/*N*. However, this will lead to the same network structure as above in the limit where $N\gg 1$, since the probability of forming reciprocal links becomes vanishingly small (~1/*N*^2^). For an example of how bow-tie architectures can also be built with finite fractions of both reciprocal and single unidrectional links see^43^.

All structural and dynamical quantities presented are averaged over the above-mentioned ensemble. The time evolution of the Kuramoto and Ising models layered on each network were simulated from random initial conditions: the phase of each node *θ*_*n*_ was drawn uniformly at random between −*π* and *π* (Kuramoto model), and the spin *s*_*n*_ of each node was drawn binomially at random with probability $p=1/2$ (Ising model). All time-averaged quantities were calculated over a time window in the steady state. The natural frequencies $\omega$ in the Kuramoto model were drawn normally at random, with mean $\langle \omega \rangle =0$ and standard deviation $\sigma =1$.

The results of analytical calculations presented in Fig. 2 were obtained following standard methods (see^38^ and the references therein for further details.) In particular, for any directed uncorrelated random network with *N* nodes, and an in-degree (*q*_*in*_) out-degree (*q*_*out*_) distribution $P({q}_{in},{q}_{out})$, the fraction of SOURCE nodes *N*_SOURCE_/*N* can be calculated from the probability of randomly selecting a SOURCE node. For any node with out-degree *q*_*out*_, this is equal to the probability of selecting a node with ${q}_{in}=0$, given by $P({q}_{in}=0,{q}_{out})$, and that at least one of its out-links leads to the CORE, given by $1-{y}_{c}^{{q}_{out}}$, where *y*_*c*_ is the probability that an out-link leads to a finite component. Taking into account all possible degrees,

10 $$\frac{{N}_{{\rm{SOURCE}}}}{N}=\sum _{{q}_{out}}\,P({q}_{in}=0,{q}_{out})\,(1-{y}_{c}^{{q}_{out}}),$$

where *y*_*c*_ can be determined self-consistently^38^. The average number of IN-CORE links can also be calculated using this formalism, considering a node randomly selected with probability $P({q}_{in},{q}_{out})$, and the probability ${C}_{m}^{{q}_{in}}{x}_{c}^{m}{(1-{x}_{c})}^{{q}_{in}-m}$ that it receives *m* in-links from nodes in finite components, given its in-degree, the probability that an in-link comes from a finite component *x*_*c*_^38^, and the probability that at least one out-link leads to a CORE node $1-{y}_{c}^{{q}_{out}}$. To ensure that such a node belongs to the CORE, it is sufficient to ensure its *m* in-links from the IN component account at most for *q*_*i*_ − 1 of its total in-links, i.e. that at least one of its in-links is from another CORE node. The average number of links $\langle m\rangle$ may then be explicitly calculated, and the number of IN-CORE links ${L}_{{\rm{IN}}\mbox{--}{\rm{CORE}}}=N\langle m\rangle$. Summing over *m* (with some in-between simplifications),

11 $${L}_{{\rm{IN}}\mbox{--}{\rm{CORE}}}=N\,\sum _{{q}_{in},{q}_{out}}\,{q}_{in}{x}_{c}P({q}_{in},{q}_{out})\,(1-{y}_{c}^{{q}_{out}})\,(1-{x}_{c}^{{q}_{in}-1}),$$

where *x*_*c*_, like *y*_*c*_, is also determined self-consistently.
